# Lanthanite-(Nd), Nd_2_(CO_3_)_3_·8H_2_O

**DOI:** 10.1107/S1600536813003164

**Published:** 2013-02-06

**Authors:** Shaunna M. Morrison, Marcelo B. Andrade, Michelle D. Wenz, Kenneth J. Domanik, Robert T. Downs

**Affiliations:** a Department of Geosciences, University of Arizona, 1040 E. 4th Street, Tucson, Arizona 85721-0077, USA; b Lunar and Planetary Laboratory, University of Arizona, 1629 E. University Blvd, Tucson, AZ. 85721-0092, USA

## Abstract

Lanthanite-(Nd), ideally Nd_2_(CO_3_)_3_·8H_2_O [dineodymium(III) tricarbonate octa­hydrate], is a member of the lanthanite mineral group characterized by the general formula *REE*_2_(CO_3_)_3_·8H_2_O, where *REE* is a 10-coordinated rare earth element. Based on single-crystal X-ray diffraction of a natural sample from Mitsukoshi, Hizen-cho, Karatsu City, Saga Prefecture, Japan, this study presents the first structure determination of lanthanite-(Nd). Its structure is very similar to that of other members of the lanthanite group. It is composed of infinite sheets made up of corner- and edge-sharing of two NdO_10_-polyhedra (both with site symmetry ..2) and two carbonate triangles (site symmetries ..2 and 1) parallel to the *ab* plane, and stacked perpendicular to *c*. These layers are linked to one another only through hydrogen bonding involving the water mol­ecules.

## Related literature

For background to the lanthanite mineral group, see: Berzelius (1825 ▶); Blake (1853 ▶); Coutinho (1955 ▶); Shinn & Eick (1968 ▶); Ansell *et al.* (1976 ▶); Dal Negro *et al.* (1977 ▶); Cesbron *et al.* (1979 ▶); Roberts *et al.* (1980 ▶); Fujimori (1981 ▶); Svisero & Mascarenhas (1981 ▶); Nagashima *et al.* (1986 ▶); Atencio *et al.* (1989 ▶); Coimbra *et al.* (1989 ▶); Graham *et al.* (2007 ▶). For information on dawsonite, see: Corazza *et al.* (1977 ▶). For details of rigid-body motion, see: Downs *et al.* (1992 ▶). For resources for bond-valence calculations, see: Brese & O’Keeffe (1991 ▶).

## Experimental

### 

#### Crystal data

Nd_2_(CO_3_)_3_·8H_2_O
*M* *_r_* = 612.64
Orthorhombic, 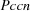

*a* = 8.9391 (4) Å
*b* = 9.4694 (4) Å
*c* = 16.9374 (8) Å
*V* = 1433.72 (11) Å^3^
*Z* = 4
Mo *K*α radiation
μ = 7.25 mm^−1^
*T* = 296 K
0.20 × 0.18 × 0.02 mm

#### Data collection

Bruker APEXII CCD area-detector diffractometer
Absorption correction: multi-scan (*SADABS*; Sheldrick, 2005 ▶) *T*_min_ = 0.325, *T*_max_ = 0.869
9235 measured reflections
1570 independent reflections
1373 reflections with *I* > 2σ(*I*)
*R*_int_ = 0.018

#### Refinement

*R*[*F* ^2^ > 2σ(*F* ^2^)] = 0.020
*wR*(*F* ^2^) = 0.057
*S* = 1.12
1570 reflections
102 parameters
H-atom parameters not refined
Δρ_max_ = 0.81 e Å^−3^
Δρ_min_ = −0.59 e Å^−3^

### 

Data collection: *APEX2* (Bruker, 2004 ▶); cell refinement: *SAINT* (Bruker, 2004 ▶); data reduction: *SAINT*; program(s) used to solve structure: *SHELXS97* (Sheldrick, 2008 ▶); program(s) used to refine structure: *SHELXL97* (Sheldrick, 2008 ▶); molecular graphics: *XtalDraw* (Downs & Hall-Wallace, 2003 ▶); software used to prepare material for publication: *publCIF* (Westrip, 2010 ▶).

## Supplementary Material

Crystal structure: contains datablock(s) I, global. DOI: 10.1107/S1600536813003164/wm2719sup1.cif

Structure factors: contains datablock(s) I. DOI: 10.1107/S1600536813003164/wm2719Isup2.hkl

Additional supplementary materials:  crystallographic information; 3D view; checkCIF report

## Figures and Tables

**Table 1:** Hydrogen-bond geometry (Å)

*D*—H⋯*A*	*D*⋯*A*
O*W*1⋯O1	2.645 (4)
O*W*1⋯O*W*1^i^	2.772 (5)
O*W*1⋯O*W*4^ii^	2.792 (5)
O*W*1⋯O*W*3^iii^	2.795 (4)
O*W*2⋯O1^iv^	2.645 (3)
O*W*2⋯O*W*2^i^	2.786 (5)
O*W*2⋯O5	2.815 (4)
O*W*2⋯O*W*4^i^	2.833 (5)
O*W*3⋯O2^v^	2.626 (5)
O*W*3⋯O*W*1^iv^	2.795 (4)
O*W*3⋯O5	2.928 (4)
O*W*3⋯O*W*3^vi^	2.994 (6)
O*W*4⋯O*W*1^vii^	2.792 (5)
O*W*4⋯O*W*2^i^	2.833 (5)
O*W*4⋯O*W*4^viii^	2.909 (9)
O*W*4⋯O*W*2^i^	3.231 (6)

Symmetry codes: (i) 
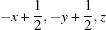
; (ii) 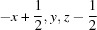; (iii) 
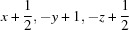
; (iv) 
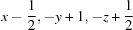
; (v) 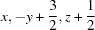; (vi) 
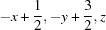
; (vii) 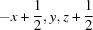; (viii) 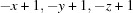.

**Fig. 1.**
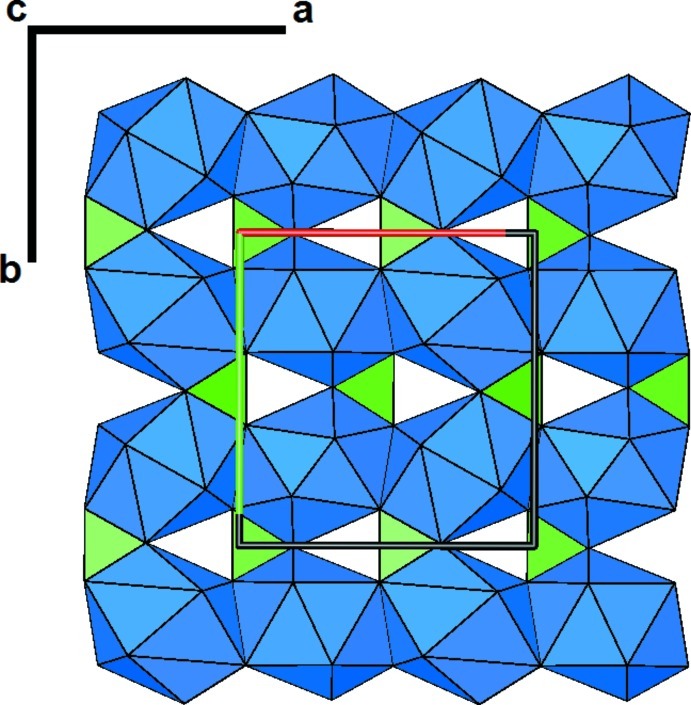
Looking down on a sheet of the lanthanite-(Nd) structure. NdO10 polyhedra are represented in blue and carbonate triangles are represented in green.

**Fig. 2.**
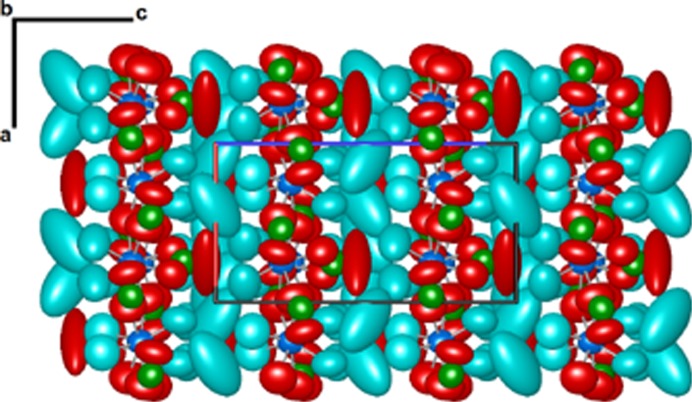
The crystal structure of lanthanite-(Nd) represented with displacement ellipsoids at the 99% probability level. Blue, green, red and cyan represent Nd, C, O atoms and H2O molecules, respectively.

**Fig. 3.**
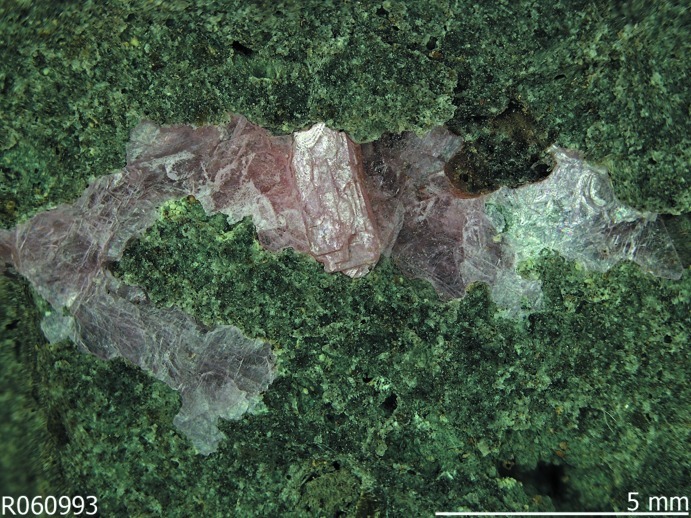
Photograph of the lanthanite-(Nd) specimen analyzed in this study, illustrating its platy habit.

## supplementary crystallographic information

### Comment

Crystals of the lanthanite group minerals exhibit a thin, platy habit and are characterized by the general formula *REE*_2_(CO_3_)_3_^.^8H_2_O, where *REE* is a 10-coordinated rare earth element. The group consists of lanthanite-(La), lanthanite-(Ce) and lanthanite-(Nd). The first of these minerals was found by Berzelius (1825) during one of his excursions to Bastnäs, Västmanland, Sweden, where he studied the local minerals and formulated the fundamentals of modern chemistry. Over 150 years later, Dal Negro *et al.* (1977) refined the crystal structure of the Bastnäs lanthanite and reported it in the non-standard setting *Pbnb* of space group No. 56. The chemistry of a different sample from this locality was later determined to be dominated by Ce (Atencio *et al.*, 1989). Of the lanthanite group minerals, the structure of lanthanite-(Ce) is the only one previously determined from a natural sample. However, Shinn & Eick (1968) synthesized lanthanite-(La) and performed a refinement in the standard setting *Pccn* of space group No. 56.

Lanthanite-(Nd) from Bethlehem, Pennsylvania, US, was first described by Blake (1853), although it was not possible to discriminate the Nd-dominance at that time. It was not until Atencio *et al.* (1989) analyzed a Bethlehem sample that it was found to be Nd-rich. Lanthanite-(Nd) has since been reported from other localities, including Curitiba, Brazil (Coutinho, 1955; Ansell *et al.*, 1976; Cesbron *et al.*, 1979; Roberts *et al.*, 1980; Fujimori, 1981; Svisero & Mascarenhas, 1981); Saga Prefecture, Japan (Nagashima *et al.*, 1986); Santa Isabel, São Paulo, Brazil (Coimbra *et al.*, 1989); and Whitianga, Coromandel Peninsula, New Zealand (Graham *et al.*, 2007). With the exception of those found in Whitianga, all lanthanite-(Nd) samples from these localities, including the one used in this study, exhibit a predominance of Nd with sub-equal La and a notable depletion of Ce. In reference to this phenomenon, Atencio *et al.* (1989) stated that lanthanite minerals comprise two distinct groups: one in which the proportions of La, Ce and Nd are similar and another in which La and Nd are similar in abundance while Ce is severely depleted or entirely absent. This trend presumably stems from differences in formational conditions, but the exact mechanism(*s*) remain(*s*) unclear.

Many of the studies referenced above reported lanthanite-(Nd) unit-cell parameters, but none reported the crystal structure. This study presents the first crystal structure refinement of lanthanite-(Nd). In the course of identifying minerals for the RRUFF project (http://rruff.info), we found an un-twinned lanthanite-(Nd) sample from Mitsukoshi, Hizen-cho, Karatsu City, Saga Prefecture, Japan and performed single-crystal X-ray diffraction.

The general structure feature of lanthanite-(Nd) is that of infinite sheets of corner- and edge-sharing NdO_10_- and carbonate-polyhedra (Fig. 1) parallel to the *ab* plane, and stacked perpendicular to *c*. The layers are linked to one another only by hydrogen bonding between water molecules (Fig. 2). This accounts for the micaceous cleavage of the lanthanite minerals (Fig. 3) (Dal Negro *et al.*, 1977). There are two distinct Nd-sites (Nd1 and Nd2 at Wyckoff positions 4 *c* and 4 *d*), as well as two C-sites (C1 and C2 at Wyckoff positions 4 *d* and 8 *e*). Nd1 and Nd2 share an edge (O3 and O4) in the *a*-direction, forming a chain. The chains are linked in the *b*-direction by NdO_10_ polyhedra sharing a corner (O5) and sharing edges with the C2 carbonate group. Nd1 is bonded to two water molecules (OW1 and OW2) while Nd2 is bonded to one (OW3) that protrude from the primary sheet in the *c*-direction. The C1 carbonate group also projects from the sheet along *c*. According to bond valence calculations (Brese & O'Keeffe, 1991), without accounting for hydrogen bonding, each O-atom of the C1 carbonate group is under-bonded, with bond valence sums of 1.64 and 1.47 bond valence units for O1 and O2, respectively. The two O1 atoms are bonded only to Nd2 and C1, resulting in an underbonding that is satisfied by hydrogen bonding as an acceptor of OW1 and OW2 (Table 1). The apex O-atom, O2, is not bonded to any cation other than C1 and therefore has much larger thermal displacement parameters and a shorter bond length (1.243 (8) Å) than usually found in carbonate groups. The C1—O2 bond is close to satisfying the rigid-body criteria of equal displacement amplitudes of C1 and O2 along the C1—O2 bond direction. The bond length, corrected for rigid-body motion is 1.268 Å (Downs *et al.*, 1992). O2 is also the acceptor of an hydrogen bond from the OW3 atom of the adjacent sheet, thus connecting the two sheets. There are not many minerals with dangling O atoms in CO_3_ groups, but these features are also observed in the crystal structures of isotypic lanthanite-(La) (Shinn & Eick, 1968), lanthanite-(Ce) (Dal Negro *et al.*, 1977) and in dawsonite, NaAlCO_3_(OH)_2_ (Corazza *et al.*, 1977). The last water molecule, OW4, is not bonded to any cation, but instead is situated between the OW1 of a given polyhedral layer and OW2 of the adjacent layer, linking the two layers together through hydrogen bonds.

### Experimental

The lanthanite-(Nd) specimen used in this study was from Mitsukoshi, Hizen-cho, Karatsu City, Saga Prefecture, Japan, and is in the collection of the RRUFF project (deposition No. R060993; http://rruff.info). The experimental empirical formula, (Nd_0.95_La_0.61_Pr_0.17_Sm_0.12_Gd_0.08_Y_0.04_Eu_0.03_)_Σ__=2_(CO_3_)_3_^.^7.97H_2_O, was based on 17 O atoms and was determined from data of a CAMECA SX100 electron microprobe at the conditions of 15 keV, 10 nA, and a beam size of 20 µm. An average of 23 analysis points yielded (wt. %): H_2_O 23.50 (by difference), CO_2_ 21.60, Y_2_O_3_ 0.70, La_2_O_3_ 16.32, Pr_2_O_3_ 4.56, Nd_2_O_3_ 26.06, Sm_2_O_3_ 3.49, Eu_2_O_3_ 0.87, Gd_2_O_3_ 2.40, Tb_2_O_3_ 0.12, Dy_2_O_3_ 0.45.

### Refinement

Due to similar X-ray scattering lengths, all rare earth elements were treated as Nd. The highest residual peak in the difference Fourier maps was located at (1/4, 3/4, 0.3413), 1.03 Å from Nd2, and the deepest hole at (0.2515, 0.8358, 0.2813), 0.81 Å from Nd2. H-atoms from water molecules could not be assigned reliably and were excluded from refinement.

### Crystal data

**Table:**

Nd_2_(CO_3_)_3_·8H_2_O	*F*(000) = 1160
*M**_r_* = 612.64	*D*_x_ = 2.838 Mg m^−^^3^
Orthorhombic, *P**c**c**n*	Mo *K*α radiation, λ = 0.71073 Å
Hall symbol: -P 2ab 2ac	Cell parameters from 6318 reflections
*a* = 8.9391 (4) Å	θ = 2.3–27.5°
*b* = 9.4694 (4) Å	µ = 7.25 mm^−^^1^
*c* = 16.9374 (8) Å	*T* = 296 K
*V* = 1433.72 (11) Å^3^	Platy, pink
*Z* = 4	0.20 × 0.18 × 0.02 mm

### Data collection

**Table:**

Bruker APEXII CCD area-detector diffractometer	1570 independent reflections
Radiation source: fine-focus sealed tube	1373 reflections with *I* > 2σ(*I*)
Graphite monochromator	*R*_int_ = 0.018
φ and ω scan	θ_max_ = 27.5°, θ_min_ = 3.2°
Absorption correction: multi-scan (*SADABS*; Sheldrick, 2005)	*h* = −11→11
*T*_min_ = 0.325, *T*_max_ = 0.869	*k* = −10→11
9235 measured reflections	*l* = −21→19

### Refinement

**Table:**

Refinement on *F*^2^	Primary atom site location: structure-invariant direct methods
Least-squares matrix: full	Secondary atom site location: difference Fourier map
*R*[*F*^2^ > 2σ(*F*^2^)] = 0.020	H-atom parameters not refined
*wR*(*F*^2^) = 0.057	*w* = 1/[σ^2^(*F*_o_^2^) + (0.0225*P*)^2^ + 5.4801*P*] where *P* = (*F*_o_^2^ + 2*F*_c_^2^)/3
*S* = 1.12	(Δ/σ)_max_ = 0.001
1570 reflections	Δρ_max_ = 0.81 e Å^−^^3^
102 parameters	Δρ_min_ = −0.59 e Å^−^^3^
0 restraints	

### Special details

**Table:**

Geometry. All e.s.d.'s (except the e.s.d. in the dihedral angle between two l.s. planes) are estimated using the full covariance matrix. The cell e.s.d.'s are taken into account individually in the estimation of e.s.d.'s in distances, angles and torsion angles; correlations between e.s.d.'s in cell parameters are only used when they are defined by crystal symmetry. An approximate (isotropic) treatment of cell e.s.d.'s is used for estimating e.s.d.'s involving l.s. planes.
Refinement. Refinement of *F*^2^ against ALL reflections. The weighted *R*-factor *wR* and goodness of fit *S* are based on *F*^2^, conventional *R*-factors *R* are based on *F*, with *F* set to zero for negative *F*^2^. The threshold expression of *F*^2^ > σ(*F*^2^) is used only for calculating *R*-factors(gt) *etc*. and is not relevant to the choice of reflections for refinement. *R*-factors based on *F*^2^ are statistically about twice as large as those based on *F*, and *R*- factors based on ALL data will be even larger.

### Fractional atomic coordinates and isotropic or equivalent isotropic displacement parameters (Å^2^)

**Table:**

	*x*	*y*	*z*	*U*_iso_*/*U*_eq_	
Nd1	0.2500	0.2500	0.250082 (15)	0.01450 (9)	
Nd2	0.2500	0.7500	0.280450 (15)	0.01381 (9)	
C1	0.2500	0.7500	0.1061 (3)	0.0211 (11)	
C2	0.4597 (4)	0.4972 (3)	0.28322 (19)	0.0142 (6)	
O1	0.3229 (3)	0.6575 (3)	0.14676 (14)	0.0181 (5)	
O2	0.2500	0.7500	0.0327 (3)	0.069 (2)	
O3	0.0234 (3)	0.3837 (3)	0.20815 (16)	0.0210 (5)	
O4	0.0234 (3)	0.6160 (3)	0.23732 (16)	0.0216 (5)	
O5	0.3158 (3)	0.4931 (3)	0.29412 (15)	0.0192 (5)	
OW1	0.3170 (3)	0.3820 (3)	0.12154 (16)	0.0232 (5)	
OW2	0.1140 (3)	0.3219 (3)	0.37844 (16)	0.0261 (6)	
OW3	0.1241 (3)	0.6457 (3)	0.40530 (17)	0.0343 (7)	
OW4	0.3878 (6)	0.3889 (5)	0.4972 (3)	0.0749 (15)	

### Atomic displacement parameters (Å^2^)

**Table:**

	*U*^11^	*U*^22^	*U*^33^	*U*^12^	*U*^13^	*U*^23^
Nd1	0.01417 (13)	0.01111 (16)	0.01822 (16)	0.00172 (9)	0.000	0.000
Nd2	0.01354 (13)	0.01037 (16)	0.01752 (16)	−0.00126 (9)	0.000	0.000
C1	0.018 (2)	0.022 (3)	0.023 (3)	0.003 (2)	0.000	0.000
C2	0.0165 (15)	0.0119 (17)	0.0143 (14)	0.0004 (12)	−0.0013 (12)	0.0014 (12)
O1	0.0181 (11)	0.0150 (12)	0.0212 (12)	0.0022 (9)	−0.0017 (9)	−0.0018 (10)
O2	0.095 (5)	0.094 (5)	0.017 (2)	0.070 (4)	0.000	0.000
O3	0.0224 (12)	0.0121 (13)	0.0284 (13)	0.0055 (10)	−0.0016 (10)	0.0001 (10)
O4	0.0188 (11)	0.0131 (14)	0.0329 (14)	−0.0039 (10)	−0.0022 (10)	−0.0029 (10)
O5	0.0128 (11)	0.0165 (13)	0.0284 (13)	0.0006 (9)	0.0027 (10)	0.0008 (10)
OW1	0.0280 (13)	0.0159 (13)	0.0255 (13)	−0.0007 (11)	0.0012 (11)	−0.0012 (10)
OW2	0.0171 (12)	0.0357 (17)	0.0254 (13)	−0.0010 (11)	0.0007 (10)	0.0006 (12)
OW3	0.0303 (15)	0.050 (2)	0.0228 (13)	−0.0153 (14)	0.0008 (11)	−0.0011 (13)
OW4	0.089 (3)	0.077 (3)	0.059 (3)	−0.028 (3)	0.035 (2)	−0.029 (2)

### Geometric parameters (Å, º)

**Table:**

Nd1—O5	2.491 (2)	Nd2—O5	2.513 (2)
Nd1—O5^i^	2.491 (2)	Nd2—O1^iv^	2.514 (2)
Nd1—O3^i^	2.492 (2)	Nd2—O1	2.514 (2)
Nd1—O3	2.492 (2)	Nd2—OW3^iv^	2.591 (3)
Nd1—OW1^i^	2.581 (3)	Nd2—OW3	2.591 (3)
Nd1—OW1	2.581 (3)	Nd2—O3^ii^	2.759 (3)
Nd1—OW2	2.582 (3)	Nd2—O3^v^	2.759 (3)
Nd1—OW2^i^	2.582 (3)	Nd2—C1	2.953 (6)
Nd1—O4^ii^	2.762 (3)	Nd2—C2^iv^	3.040 (3)
Nd1—O4^iii^	2.762 (3)	C1—O2	1.243 (8)
Nd1—C2	3.051 (3)	C1—O1	1.291 (4)
Nd1—C2^i^	3.051 (3)	C1—O1^iv^	1.291 (4)
Nd2—O4	2.499 (3)	C2—O4^ii^	1.263 (4)
Nd2—O4^iv^	2.499 (3)	C2—O3^ii^	1.272 (4)
Nd2—O5^iv^	2.513 (2)	C2—O5	1.300 (4)
			
O5—Nd1—O5^i^	145.15 (12)	O4^iv^—Nd2—O5	109.18 (8)
O5—Nd1—O3^i^	111.28 (8)	O5^iv^—Nd2—O5	169.43 (12)
O5^i^—Nd1—O3^i^	78.92 (8)	O4—Nd2—O1^iv^	72.75 (8)
O5—Nd1—O3	78.92 (8)	O4^iv^—Nd2—O1^iv^	76.70 (8)
O5^i^—Nd1—O3	111.28 (8)	O5^iv^—Nd2—O1^iv^	71.64 (8)
O3^i^—Nd1—O3	146.88 (12)	O5—Nd2—O1^iv^	118.74 (8)
O5—Nd1—OW1^i^	139.03 (8)	O4—Nd2—O1	76.70 (8)
O5^i^—Nd1—OW1^i^	75.53 (8)	O4^iv^—Nd2—O1	72.75 (8)
O3^i^—Nd1—OW1^i^	72.68 (8)	O5^iv^—Nd2—O1	118.74 (8)
O3—Nd1—OW1^i^	79.45 (8)	O5—Nd2—O1	71.64 (8)
O5—Nd1—OW1	75.53 (8)	O1^iv^—Nd2—O1	51.48 (11)
O5^i^—Nd1—OW1	139.03 (8)	O4—Nd2—OW3^iv^	141.63 (9)
O3^i^—Nd1—OW1	79.45 (8)	O4^iv^—Nd2—OW3^iv^	72.13 (9)
O3—Nd1—OW1	72.68 (8)	O5^iv^—Nd2—OW3^iv^	69.98 (9)
OW1^i^—Nd1—OW1	64.96 (12)	O5—Nd2—OW3^iv^	101.07 (10)
O5—Nd1—OW2	67.38 (8)	O1^iv^—Nd2—OW3^iv^	135.64 (9)
O5^i^—Nd1—OW2	83.13 (9)	O1—Nd2—OW3^iv^	139.06 (9)
O3^i^—Nd1—OW2	139.15 (9)	O4—Nd2—OW3	72.13 (9)
O3—Nd1—OW2	73.95 (8)	O4^iv^—Nd2—OW3	141.63 (9)
OW1^i^—Nd1—OW2	136.78 (8)	O5^iv^—Nd2—OW3	101.07 (10)
OW1—Nd1—OW2	133.78 (8)	O5—Nd2—OW3	69.98 (9)
O5—Nd1—OW2^i^	83.13 (9)	O1^iv^—Nd2—OW3	139.06 (9)
O5^i^—Nd1—OW2^i^	67.38 (8)	O1—Nd2—OW3	135.64 (9)
O3^i^—Nd1—OW2^i^	73.95 (8)	OW3^iv^—Nd2—OW3	70.59 (13)
O3—Nd1—OW2^i^	139.15 (9)	O4—Nd2—O3^ii^	120.36 (9)
OW1^i^—Nd1—OW2^i^	133.78 (8)	O4^iv^—Nd2—O3^ii^	62.32 (9)
OW1—Nd1—OW2^i^	136.78 (8)	O5^iv^—Nd2—O3^ii^	130.16 (8)
OW2—Nd1—OW2^i^	65.29 (12)	O5—Nd2—O3^ii^	48.87 (7)
O5—Nd1—O4^ii^	48.95 (8)	O1^iv^—Nd2—O3^ii^	116.91 (8)
O5^i^—Nd1—O4^ii^	127.62 (8)	O1—Nd2—O3^ii^	70.95 (8)
O3^i^—Nd1—O4^ii^	62.36 (9)	OW3^iv^—Nd2—O3^ii^	74.51 (9)
O3—Nd1—O4^ii^	120.53 (9)	OW3—Nd2—O3^ii^	98.79 (9)
OW1^i^—Nd1—O4^ii^	119.54 (8)	O4—Nd2—O3^v^	62.32 (9)
OW1—Nd1—O4^ii^	68.74 (8)	O4^iv^—Nd2—O3^v^	120.36 (9)
OW2—Nd1—O4^ii^	103.32 (8)	O5^iv^—Nd2—O3^v^	48.87 (7)
OW2^i^—Nd1—O4^ii^	68.87 (8)	O5—Nd2—O3^v^	130.16 (8)
O5—Nd1—O4^iii^	127.62 (8)	O1^iv^—Nd2—O3^v^	70.95 (8)
O5^i^—Nd1—O4^iii^	48.95 (8)	O1—Nd2—O3^v^	116.91 (8)
O3^i^—Nd1—O4^iii^	120.53 (9)	OW3^iv^—Nd2—O3^v^	98.79 (9)
O3—Nd1—O4^iii^	62.36 (9)	OW3—Nd2—O3^v^	74.51 (9)
OW1^i^—Nd1—O4^iii^	68.74 (8)	O3^ii^—Nd2—O3^v^	171.97 (11)
OW1—Nd1—O4^iii^	119.54 (8)	O2—C1—O1	122.2 (2)
OW2—Nd1—O4^iii^	68.87 (8)	O2—C1—O1^iv^	122.2 (2)
OW2^i^—Nd1—O4^iii^	103.32 (8)	O1—C1—O1^iv^	115.5 (5)
O4^ii^—Nd1—O4^iii^	171.14 (11)	O4^ii^—C2—O3^ii^	125.6 (3)
O4—Nd2—O4^iv^	146.01 (13)	O4^ii^—C2—O5	117.4 (3)
O4—Nd2—O5^iv^	109.18 (8)	O3^ii^—C2—O5	117.0 (3)
O4^iv^—Nd2—O5^iv^	74.05 (8)	O4^ii^—C2—O1	113.5 (2)
O4—Nd2—O5	74.05 (8)		

Symmetry codes: (i) −*x*+1/2, −*y*+1/2, *z*; (ii) *x*+1/2, −*y*+1, −*z*+1/2; (iii) −*x*, *y*−1/2, −*z*+1/2; (iv) −*x*+1/2, −*y*+3/2, *z*; (v) −*x*, *y*+1/2, −*z*+1/2.

### Hydrogen-bond geometry (Å)

**Table:**

*D*—H···*A*	*D*···*A*
O*W*1···O1	2.645 (4)
O*W*1···O*W*1^i^	2.772 (5)
O*W*1···O*W*4^vi^	2.792 (5)
O*W*1···O*W*3^ii^	2.795 (4)
O*W*2···O1^vii^	2.645 (3)
O*W*2···O*W*2^i^	2.786 (5)
O*W*2···O5	2.815 (4)
O*W*2···O*W*4^i^	2.833 (5)
O*W*3···O2^viii^	2.626 (5)
O*W*3···O*W*1^vii^	2.795 (4)
O*W*3···O5	2.928 (4)
O*W*3···O*W*3^iv^	2.994 (6)
O*W*4···O*W*1^ix^	2.792 (5)
O*W*4···O*W*2^i^	2.833 (5)
O*W*4···O*W*4^x^	2.909 (9)
O*W*4···O*W*2^i^	3.231 (6)

Symmetry codes: (i) −*x*+1/2, −*y*+1/2, *z*; (ii) *x*+1/2, −*y*+1, −*z*+1/2; (iv) −*x*+1/2, −*y*+3/2, *z*; (vi) −*x*+1/2, *y*, *z*−1/2; (vii) *x*−1/2, −*y*+1, −*z*+1/2; (viii) *x*, −*y*+3/2, *z*+1/2; (ix) −*x*+1/2, *y*, *z*+1/2; (x) −*x*+1, −*y*+1, −*z*+1.
